# Changes in RSV-associated lower respiratory tract infections among hospitalized and outpatient children under 2 years in Northern Bavaria after general recommendation of Nirsevimab immunization in 2024

**DOI:** 10.1007/s15010-025-02654-1

**Published:** 2025-10-17

**Authors:** Ying Zhou, Katharina Hecker, Géraldine Engels, Oliver Andres, Kerstin Knies, Christine Krempl, Benedikt Weissbrich, Christoph Härtel, Andrea Streng, Johannes Liese

**Affiliations:** 1 Department of Pediatrics, University Hospital of Würzburg (UKW), Josef- Schneider-Straße 2, D-97080 Würzburg, Germany; 2 Institute of Virology and Immunobiology, University of Würzburg, Versbacher Str. 7, 97078 Würzburg, Germany

**Keywords:** RSV, Immunization, Nirsevimab, LRTI, Infants, Surveillance

## Abstract

**Purpose:**

This study investigated changes in the incidence and age distribution of RSV-associated lower respiratory tract infections (RSV-LRTI) among children in Northern Bavaria after the general recommendation of Nirsevimab immunization in 2024.

**Methods:**

Postnatal Nirsevimab immunization coverage was assessed at the University Hospital Würzburg (UKW) in children born between 11/2024 and 03/2025. Age distribution of in- and outpatients < 2 years with PCR-confirmed RSV-LRTI was assessed from UKW (ICD-10 based) and pediatric practices in Würzburg for November-March in 2022/23 (S1), 2023/24 (S2) and 2024/25 (S3). Age distribution of RSV cases from nationwide mandatory laboratory RSV surveillance (Robert Koch-Institute) was analyzed for November-March in 2023/24 and 2024/25.

**Results:**

Between 11/2024 and 03/2025, postnatal Nirsevimab immunization coverage for newborns at the UKW was 68% (566/833). In the RSV seasons S1/S2/S3, 98/84/29 children < 2 years with RSV-LRTI were hospitalized. The proportion of children < 1 year decreased from 78%/80% in S1/S2 to 42% in S3 (S1 vs. S3 *p* < 0.001, S2 vs. S3 *p* < 0.001). In 36/68/30 outpatients < 2 years with RSV-LRTI, the proportion of children < 1 year decreased from 44%/60% in S1/S2 to 33% in S3 (S1 vs. S3 *p* = 0.358; S2 vs. S3 *p* = 0.014). In nationwide laboratory RSV surveillance, 23,171/12,826 children < 2 years were reported in S2/S3, with a decrease in the proportion of children < 1 year from 65% in S2 to 47% in S3 (*p* < 0.001).

**Conclusions:**

We observed a clear decrease of RSV-LRTI in hospitalized and outpatient infants < 1 year of age in the first RSV season, suggesting a relevant impact following Nirsevimab recommendation in Germany.

## Introduction

Respiratory syncytial virus (RSV) is a seasonal pathogen that predominantly affects children < 2 years. It is a leading cause of lower respiratory tract infections (LRTI) in young children and remains the most common cause for hospitalization among children < 1 year old [1]. During the 2023/24 RSV season in Germany, sentinel surveillance data estimated hospitalization rates ranging from approximately 9 to 37 per 1000 infants [2].

Nirsevimab, a monoclonal antibody, provides passive immunization against severe RSV disease in infants, offering protection for about six months after administration [3]. In October 2022, Nirsevimab was licensed in the European Union, as a single intramuscular dose for the prevention of RSV-LRTIs in newborns and infants during their first RSV season. Several European countries began implementing regional or national immunization programs for newborns and infants with Nirsevimab in September 2023. Analyses from these post-marketing programs have confirmed the effectiveness of about 80% in reducing medically attended RSV-LRTI [4].

The German Standing Committee on Vaccination (STIKO) recommended Nirsevimab for all infants under 12 months of age in June 2024. Infants born between April and September 2024 should be immunized between September and November, preceding their first RSV season, whereas those born between October and March should be immunized shortly after birth, ideally between the 3rd and 10th day of life [5]. Maternal RSV vaccination during pregnancy has not been recommended by STIKO so far. Therefore, it is usually not reimbursed by health insurances, and available mostly through the private medical sector.

To evaluate the potential impact of Nirsevimab immunization in the first season after the general recommendation, we assessed the local Nirsevimab immunization rate in newborns, and determined the age distribution among local hospitalized and outpatient children < 2 years diagnosed with RSV-LRTI in the RSV season 2024/25 and two previous seasons. In addition, the age distribution of all children < 2 years with RSV detection reported from mandatory national laboratory surveillance was compared between the season 2023/24 and 2024/25.

## Methods

### Study setting

In 2022, the region of Würzburg (Northern Bavaria/Germany) covered an annual resident population of about 5,400 children < 2 years of age, with two large children’s hospitals and about 18 pediatric practices in the area. At the University Hospital Würzburg (UKW), annually about 2,100 children are born, mainly from Würzburg and the surrounding areas.

### Data collection

#### Nirsevimab coverage

The postnatal Nirsevimab immunization coverage of all children born at the UKW in the period between November 2024 and March 2025 was determined using the internal UKW database.

#### Hospitalized children

According to internal hospital standard procedures, regular RSV testing with PCR was done in all admitted children presenting with symptoms of LRTI throughout the observation period of three RSV seasons. Children < 2 years admitted with RSV-LRTI to the UKW Department of Pediatrics in the period November to March in three RSV seasons (S1: 2022/23, S2: 2023/24, S3: 2024/25) were identified from the hospital database, using RSV-specific ICD-10 diagnoses (J12.1 RSV pneumonia; J20.5 RSV acute bronchitis; J21.0 RSV acute bronchiolitis; B97.4 RSV as cause of other diseases). All identified children were confirmed by medical chart review for RSV-positive PCR results and a diagnosis of LRTI.

#### Outpatient children

Data on LRTI outpatients originated from an ongoing surveillance study based in a network of 12 pediatric practices in Würzburg. Outpatient children < 2 years with acute febrile LRTIs, presenting at a pre-defined weekday during the period from November to March in the three RSV seasons S1-S3, underwent oropharyngeal swab testing for RSV and other respiratory viruses using multiplex PCR after parental consent. Acute LRTI signs/symptoms as retractions/flaring of nostrils, wheezing/grunting/stridor, tachypnea, dyspnea, abnormal auscultation and O_2_ saturation < 96% and LRTI diagnoses were documented by the pediatricians.

#### Children reported by laboratory surveillance (nationwide)

Data from nationwide mandatory RSV laboratory reporting (implemented since 2023) were extracted for children < 2 years of age (Robert Koch-Institute, Germany; URL: https://survstat.rki.de; by individual data extraction on request) during the period from November to March in the seasons 2023/24 (S2) and 2024/25 (S3), and the age distribution was assessed.

### Statistics

RSV cases were analyzed per season, stratified by age groups (< 6 months, 6–11 months and 12–23 months). For each data source, differences in age group distributions across seasons were evaluated using the Chi² test, both overall (*p* < 0.05 considered significant) and in pairwise comparisons, with Bonferroni correction applied to adjust for multiple testing (*p* < 0.017 considered significant). Odds ratios (OR) with corresponding 95% confidence intervals (CI) were calculated for children < 1 year of age.

## Results

### Postnatal nirsevimab coverage in the newborn cohort at university hospital Würzburg 2024/2025

From November 2024 to March 2025, 833 infants were born at the UKW. Overall postnatal Nirsevimab coverage at the UKW during this period was 67.9% (566/833) (95% CI: 64.8% – 71.1%). Monthly immunization rates were 69.4% (118/170) in November 2024, peaked at 76.7% (135/176) in January 2025 and declined to 56.5% (87/154) in March 2025 **(**Table 1**)**.

**Table 1:** Nirsevimab immunization rates for newborns at Würzburg university hospital from November 2024 to March 2025

	November 2024	December 2024	January 2025	February 2025	March 2025	November 2024 to March 2025
Immunized with Nirsevimab	118 (69.4%)	119 (68.8%)	135 (76.7%)	107 (66.9%)	87 (56.5%)	566 (67.9%)
Not immunized	52 (30.6%)	54 (31.2%)	41 (23.3%)	53 (33.1%)	67 (43.5%)	267 (32.1%)
All newborn children	170	173	176	160	154	833

Percentages represent the proportion of immunized/not-immunized newborns relative to the total of all newborn children in the respective month

### RSV-LRTIs in hospitalized children in the department of pediatrics (UKW) 2022/23-2024/25

Across the three seasons, RSV-LRTI were reported for 211 hospitalized patients (median age 5 months, IQR 2–13), with 98 children in S1 (median age 4 months, IQR 2–11), 84 in S2 (median age 5 months, IQR 2–11) and 29 in S3 (median age 15 months, IQR 5–18), respectively (*p* = 0.013). The overall median length of stay in hospital was 4 days (IQR 2–7); 28 children (13.3%) were treated in the pediatric intensive care unit. During the 3 seasons, 44.1% (93/211) were diagnosed with bronchitis, 39.8% (84/211) with bronchiolitis and 8.5% (18/211) with pneumonia.

**Fig. 1:**
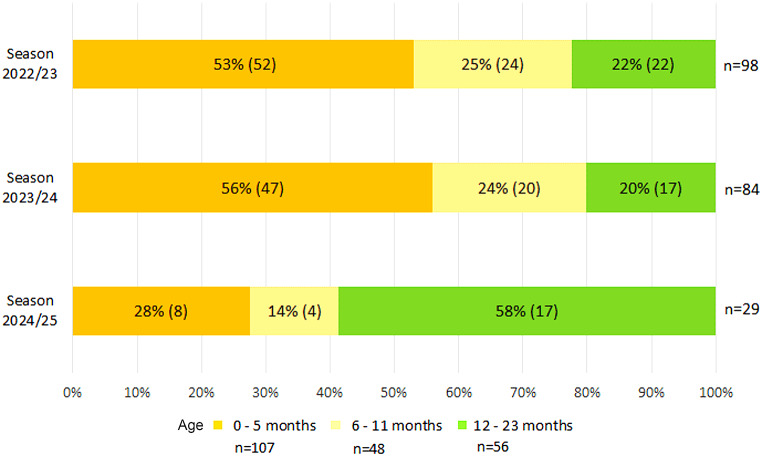
Children under 2 years of age with RSV-associated lower respiratory tract infections at the University Hospital Würzburg in 3 winter seasons (November-March) 2022/23-2024/25

In S1 and S2, 78% (76/98) and 80% (67/84) of the children with RSV-LRTI were < 1 year of age, compared with 42% (12/29) children in S3 (S3 vs. S1: *p* < 0.001; OR = 0.20, 95% CI: 0.08–0.49 and S3 vs. S2: *p* < 0.001; OR = 0.18, 95% CI: 0.07–0.44) (Fig. 1). Among the subgroup of children < 6 months, the proportion of patients decreased from 53% (52/98) in S1 and 56% (47/84) in S2 to 28% (8/29) in S3.

### RSV-LRTIs in outpatient children in 12 pediatric practices in Würzburg 2022/23-2024/25

From S1 to S3, RSV-LRTI were reported in 134 outpatient children < 2 years (median age 12 months, IQR 6–16), with 36 children in S1 (median age 12 months, IQR 6–17), 68 in S2 (median age 9 months, IQR 6–15) and 30 in S3 (median age 15 months, IQR 9–-19), respectively (*p* = 0.054). Overall, 82.1% (110/134) were diagnosed with bronchitis, 7.5% (10/134) with bronchiolitis and 3.7% (5/134) with pneumonia.

**Fig. 2:**
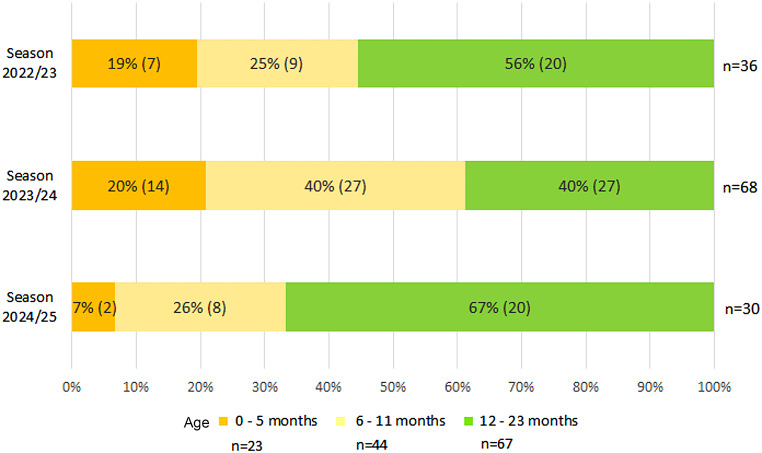
Children under 2 years of age with RSV-associated lower respiratory tract infections in 12 pediatric practices in Würzburg in 3 winter seasons (November-March) 2022/23-2024/25

In S1 and S2, 44% (16/36) and 60% (41/68) children with RSV-LRTI were < 1 year of age, compared with 33% (10/30) children in S3 (S3 vs. S2: *p* = 0.014, significant for the Bonferroni-adjusted significance level; OR = 0.33, 95% CI: 0.13–0.81) (Fig. 2). Among the subgroup of children < 6 months, the proportion of patients with RSV-LRTI decreased from 19% (7/36) in S1 and 20% (14/68) in S2 to 7% (2/30) in S3.

### RSV laboratory notifications from national mandatory reporting 2023/24-2024/25

**Fig. 3:**
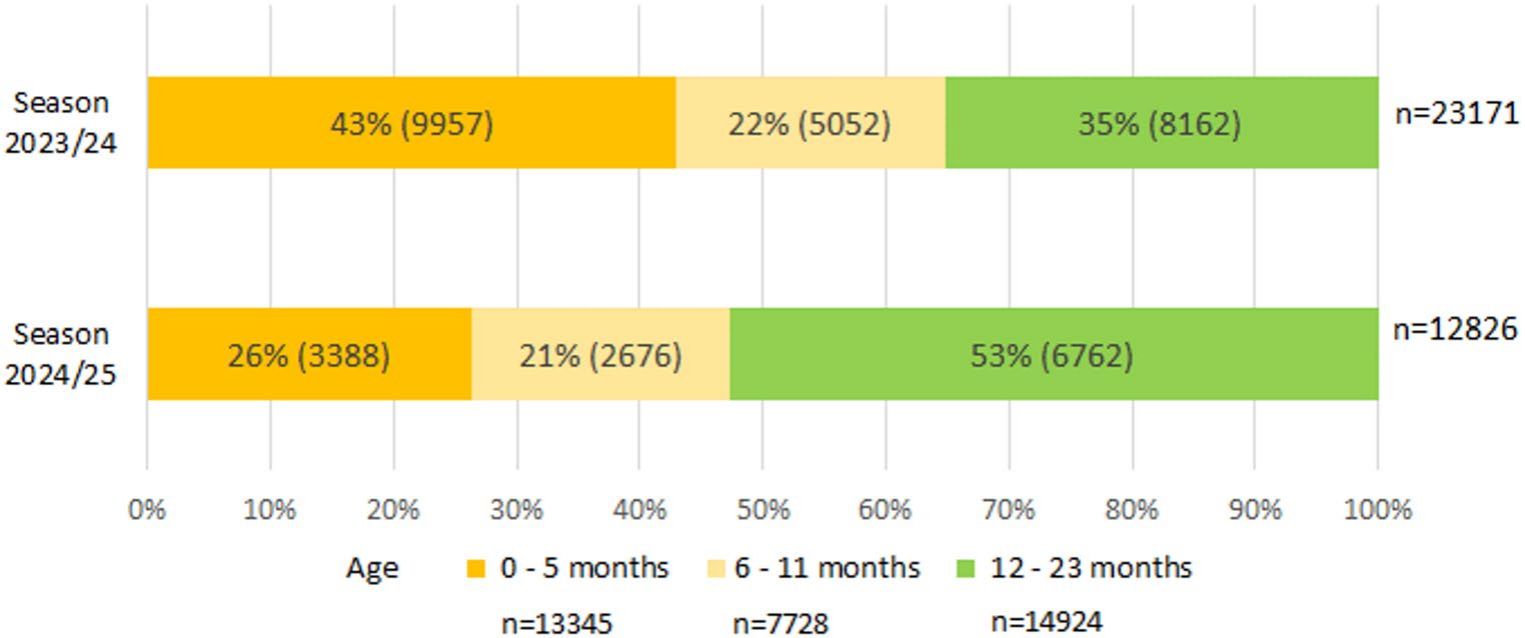
RSV detections of children under 2 years of age in the nationwide laboratory surveillance pre- and post Nirsevimab immunization recommendation in Germany (Data source: Robert Koch-Institute, Germany, https://survstat.rki.de, by individual data extraction on request. Query date: 03.07.2025)

In the nationwide mandatory laboratory surveillance implemented since 2023, a total of 23,171 RSV infections in children < 2 years of age were reported for S2 and 12,826 for S3. In S2, 65% (15,009/23,171) patients were < 1 year of age, compared with 47% (6,064/12,826) patients in S3 (*p* < 0.001; S3 vs. S2: OR = 0.49, 95% CI: 0.47–0.51). Among the subgroup of children < 6 months, the proportion of patients decreased from 43% (9,957/23,171) in S2 to 26% (3,388/12,826) in S3 (Fig. 3). The proportion of infants 6–11 months of age was 22% (S2) and 21% (S3). The proportion of children 12–23 months of age increased from 35% (S2) to 53% (S3).

## Discussion

Following the general STIKO recommendation in June 2024 to immunize all infants < 1 year of age, the monoclonal anti-RSV antibody Nirsevimab became reimbursable by statutory health insurance in September 2024 [5]. In our local study in Würzburg, the postnatal immunization coverage in newborns at the UKW in the winter season 2024/25 was 68% (95% CI: 65% − 71%), reflecting high acceptance of the STIKO recommendation. The slight decline of immunization coverage in March 2025 may be due to the reduced parental awareness, with some parents possibly assuming that the RSV season had ended.

This regional study estimated the potential impact of Nirsevimab immunization on RSV-LRTI, by comparing the incidence and proportional age distribution of RSV-LRTIs in the RSV seasons before and after the general recommendation. Compared to the first two seasons, the absolute number of children < 2 years (and < 1 year) with RSV-LRTI declined in 2024/25 among both inpatients and outpatients. The overall decline of RSV-LRTIs observed in both groups, < 1 year and 1 to < 2 years of age, was possibly due to a weaker RSV season, reflecting usual annual fluctuations in the RSV incidence. A comparatively weaker RSV season of 2024/25 was additionally suggested by a decrease in RSV laboratory reports from the nationwide laboratory surveillance, including a decrease among children aged 12–23 months who were not immunized against RSV. In our study, the large decrease in the proportion of RSV-LRTIs especially among hospitalized and outpatient infants < 6 months of age, and to a lesser extent among those < 1 year, compared to the previous two seasons suggests a positive impact of the RSV-immunization in newborn infants. In addition, our local observation is confirmed by a large data set from nationwide mandatory RSV laboratory surveillance, which showed a decrease of 66% in infants < 6 months and 47% in infants 6–11 months of age between RSV season 2023/24 and 2024/25 reported with a positive RSV test. Our results are also well comparable to an analysis of reported case data across Germany for the seasons 2023/24 and 2024/25 [6].

The findings of the present study align closely with research from other European countries, which even report a stronger impact of Nirsevimab in reducing RSV-LRTI in infants < 6 months, and to a lesser extent in those < 1 year of age, once sufficient immunization coverage of at least 80% has been obtained. A systematic review in 2025 of 32 studies from five countries (France, Italy, Luxembourg, Spain, and the United States) concluded that Nirsevimab reduced the risk of RSV-associated hospitalization by 83%, ICU admission by 81% and RSV-LRTI by 75% in infants aged 0–11 months [4]. These real-world findings were broadly consistent with efficacy data from a large controlled clinical pre-licensure study, which reported an overall efficacy of 76% in preventing RSV-associated hospitalizations among infants < 1 year of age, with country-specific rates of 90% in France, 74% in Germany and 83% in the United Kingdom [7]. It may be expected that with an increase of the immunization coverage in the coming RSV seasons in Germany, the impact on RSV LRTI incidence in infants will also continue to increase.

However, a coverage rate of approximately 70% among newborns in our hospital already reflects high parental acceptance of Nirsevimab, especially considering the relatively recent recommendation of this first widespread use of monoclonal antibodies in the general infant population. In the present study, we assessed postnatal immunizations given in our hospital to infants born during the RSV season 2024/25. Immunizations of newborns given in pediatric practices and other hospitals in the area were not available, so the ‘true’ local coverage could even be higher. Additionally, we cannot rule out that a few infants did not receive immunization because their mothers had already been immunized with an active RSV vaccine during pregnancy, which, however, is neither generally recommended nor reimbursed in Germany. Coverage is likely to increase in the coming seasons as efforts to address potential parental concerns about the safety and efficacy of this new prevention strategy and awareness of RSV-associated risks will become more widespread.

The study was not designed to evaluate the effectiveness of Nirsevimab, and can therefore only provide indirect insights into its potential effects. The limitations of our study include the geographic restriction to the Würzburg area and the relatively small number of hospitalized and outpatient RSV-LRTIs, especially in season 2024/25. In the regional network of pediatric practices, the ‘real’ burden of RSV-LRTI would be expected to be about 4–5 times higher than reflected by the presented absolute numbers, since RSV testing in the frame of our study was done only on one day per week. Nevertheless, the regionally observed decrease in hospitalized and non-hospitalized infants with RSV-LRTI was consistent with decreases in the incidence and changes in the age distribution of RSV reported cases derived from national mandatory RSV laboratory surveillance.

In conclusion, this is one of the first studies from Germany to document a decline of both virologically and clinically confirmed RSV-LRTI in the winter season 2024/25 in children < 1 year of age. This decline suggests a relevant impact of the successful implementation of Nirsevimab immunization in Germany after the national recommendation for all newborns.

## Data Availability

Data on in- and outpatients from Würzburg are not publicly available. Data from SurvStat (Robert Koch-Institute) are available in aggregated form, from [https://survstat.rki.de/](https:/survstat.rki.de) by individual data extraction on request.
